# Supplementary material for: Semi-field and surveillance data define the natural diapause timeline for Culex pipiens across the United States
Source: Commun Biol. 2022 Nov 27;5:1300. doi: 10.1038/s42003-022-04276-x (PMC9701209; doi:10.1038/s42003-022-04276-x)
Supplement: Supplementary file 2 — Supplementary Information [file 42003_2022_4276_MOESM2_ESM.pdf]

Supporting Information

Semi-field and surveillance data define the natural diapause timeline for *Culex pipiens* across the United States

Eleanor N. Field[1], John J. Shepard[2], Mark E. Clifton[3], Keith J. Price[4], Bryn J. Witmier[4], Kirk Johnson[5], Broox Boze[6], Charles Abadam[7], Gregory D. Ebel[8], Philip M. Armstrong[2], Christopher M. Barker[9], Ryan C. Smith[1,*]

[1]Department of Entomology, Iowa State University, Ames, Iowa 50011

[2]Department of Environmental Sciences, Connecticut Agricultural Experiment Station, New Haven, Connecticut 06511

[3]North Shore Mosquito Abatement District, Northfield, Illinois 60093

[4]Pennsylvania Department of Environmental Protection, Harrisburg, Pennsylvania 17110

[5]Metropolitan Mosquito Control District, St. Paul, Minnesota 55104

[6]Vector Disease Control International, Broomfield, CO 80020

[7]Suffolk Mosquito Control District, Suffolk, Virginia 23234

[8]Department of Microbiology, Immunology and Pathology, Colorado State University, Ft. Collins, Colorado 80523

[9]Department of Pathology, Microbiology and Immunology, School of Veterinary Medicine, University of California, Davis, Davis, California 95616

*Corresponding author: smithr@iastate.edu

Supplemental Figures

Figure S1. Detection of autogenous mosquitoes in the *Culex pipiens* lab colony.

Figure S2. Description of sites used for semi-field studies of mosquito diapause.

Figure S3. Temperature and photoperiod associated with semi-field experimental cohorts.

Figure S4. Evaluating pupal exposure to environmental signals under semi-field conditions.

Figure S5. Temperature comparisons of 2020 and 2021 to 30-year averages.

Figure S6. Seasonal changes to diurnal temperature fluctuations.

Figure S7. Mosquito surveillance sites in Iowa.

Figure S8. Gravid populations are distinct from general *Cx. pipiens* mosquito abundance.

Figure S9. Geographic locations and elevations of trapping site locations across the United States.

Figure S10. End-of-season temperature and photoperiod conditions at trapping sites across the United States.

Supplemental Tables

Table S1: Larval Culex pipiens semi-field group descriptions, Ames IA.

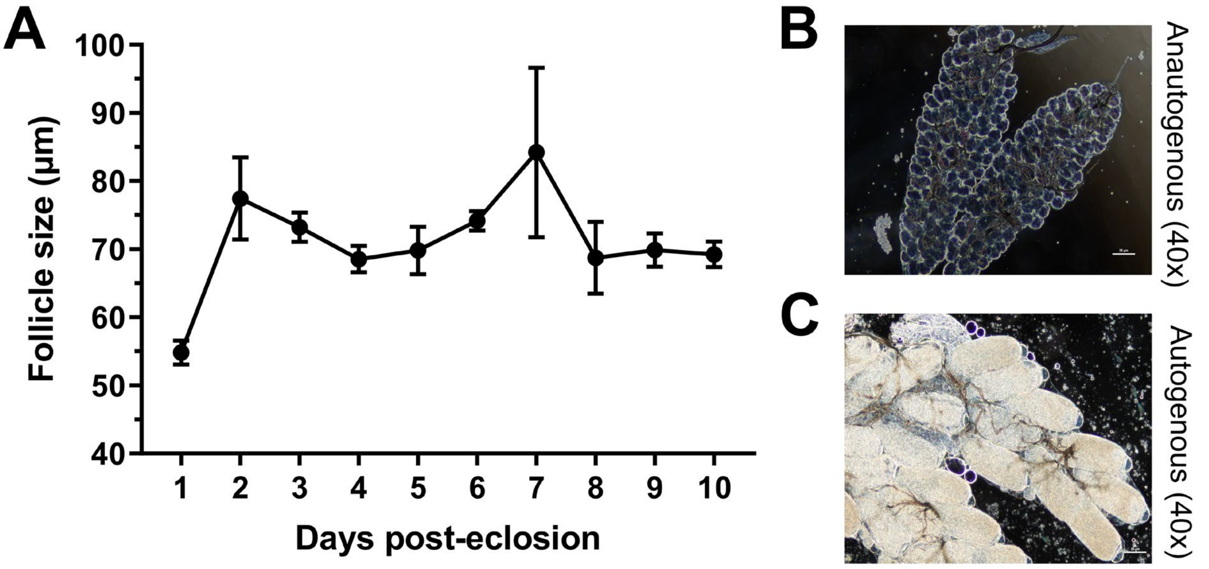

Figure S1. Detection of autogenous mosquitoes in the *Culex pipiens* lab colony. Adult female *Cx. pipiens* were dissected daily from 1 to 10 days post-eclosion to examine ovarian development and average follicle size (**A**). A total of 10 mosquitoes were used to calculate the average follicle size for days 1-4, while 4 mosquitoes were used for days 5-10. Example images display “normal” anautogenous ovaries (**B**) or less frequent examples of autogenous mosquito ovaries (**C**) as evidenced by the elongation and granulation of the follicle in the absence of a blood meal. Images are displayed with 40x magnification.

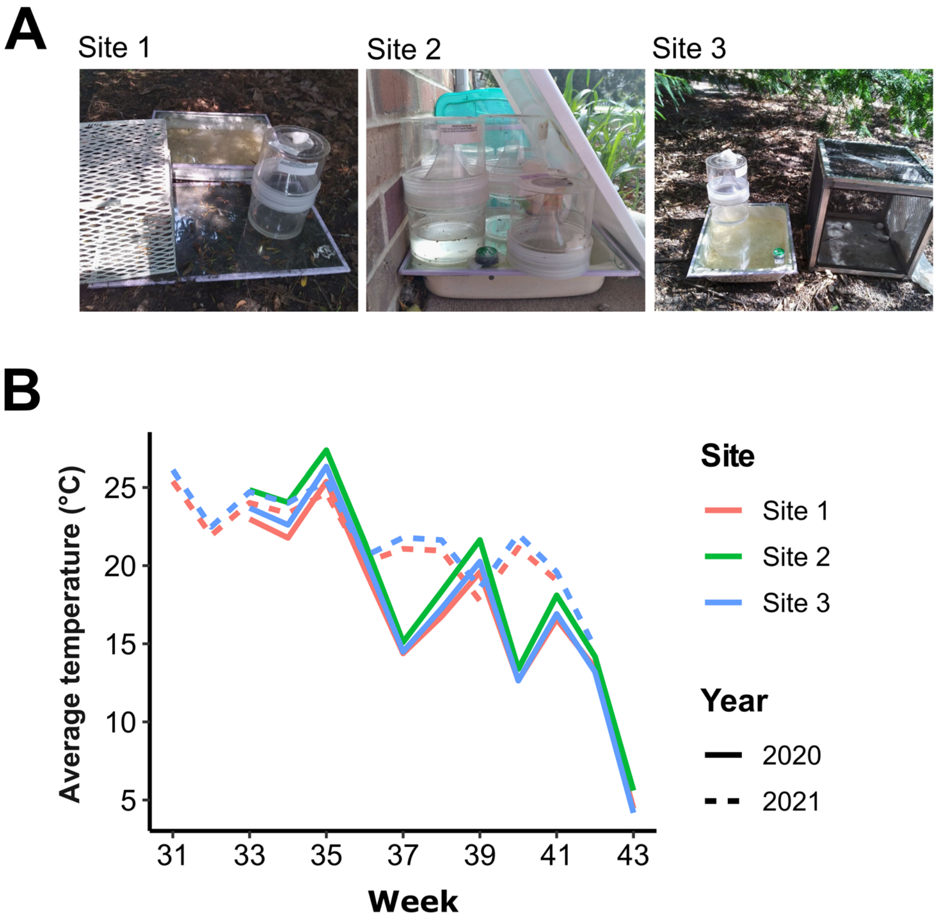

Figure S2. Descriptions and temperatures of sites used in semi-field studies. Three site locations were selected in 2020 in Ames, IA (USA) for semi-field studies to examine mosquito diapause induction (**A**). Sites 1 and 3 were used again in 2021 for the second year of the study. (**B**) Temperatures were recorded at each site using Hobo MX2202 data loggers to evaluate any differences in temperature in the microenvironment.

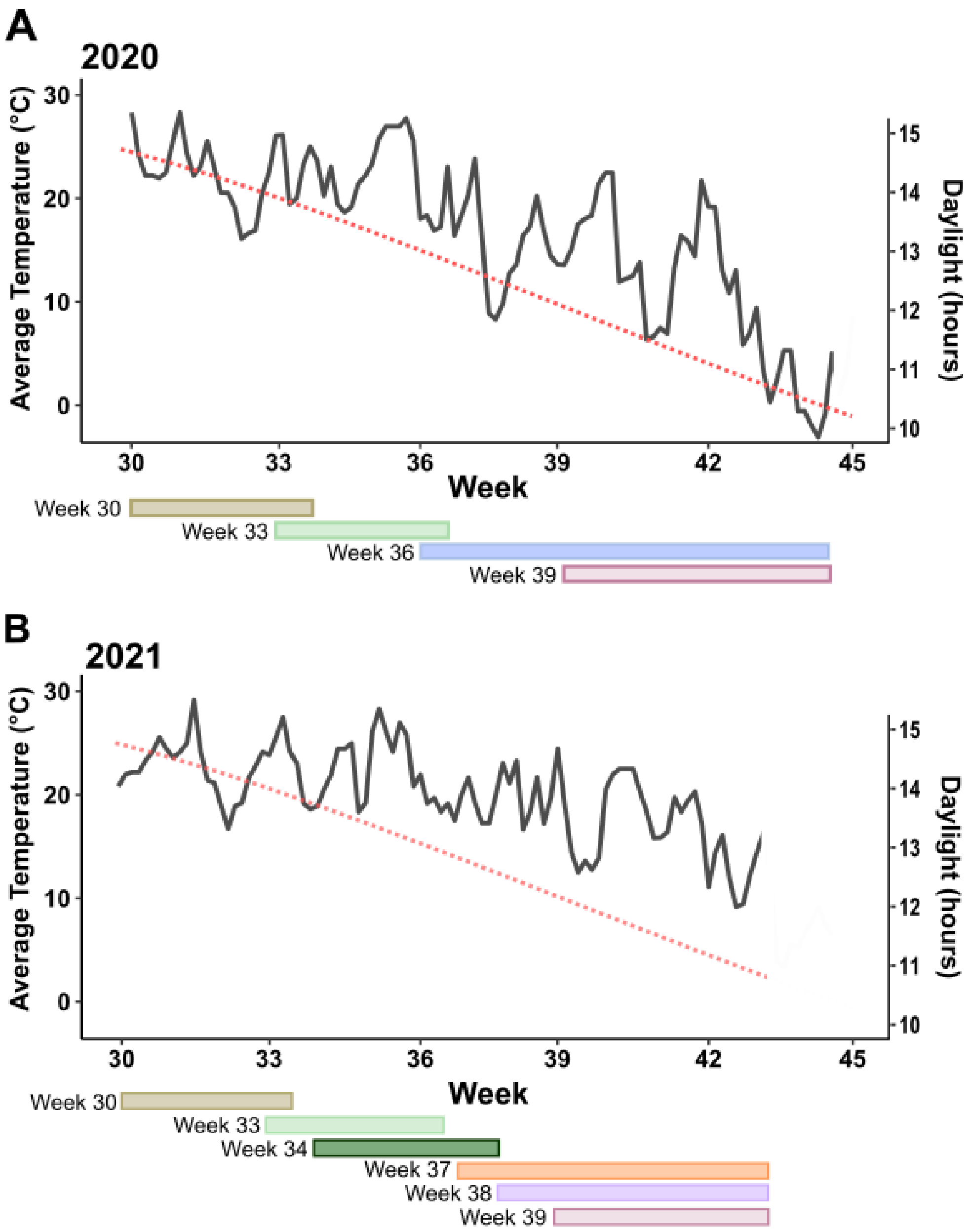

Figure S3. Temperature and photoperiod associated with semi-field experimental cohorts. Temperature (solid line) and photoperiod (dotted line) values are displayed for the experimental cohorts used in semi-field experiments for 2020 (**A**) and 2021 (**B**). For each year of experiments, larval cohorts are denoted below with the time of field placement (week) to the last adult eclosion or death. The length of bar represents the time in which each cohort was active.

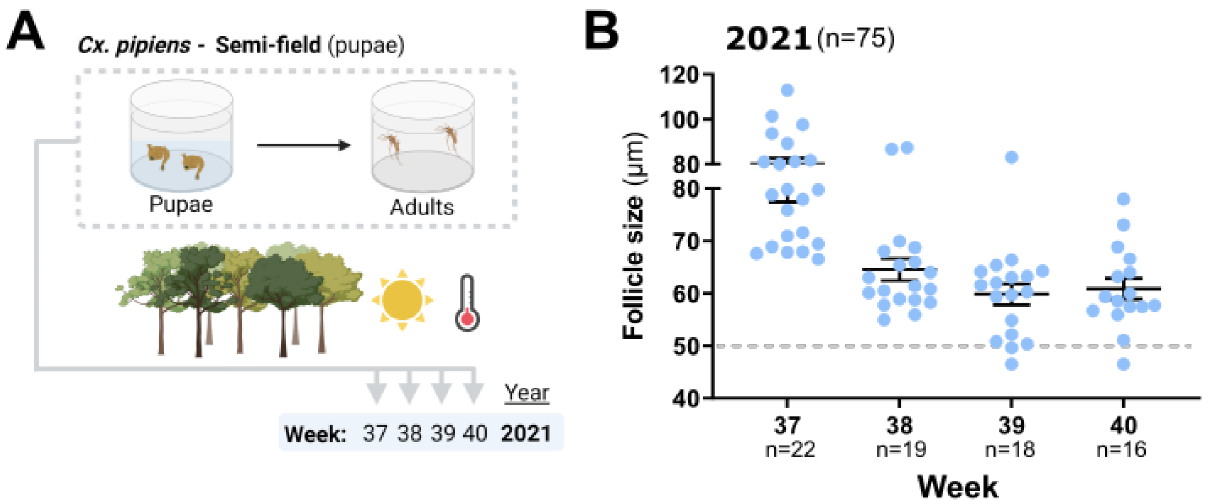

Figure S4. Evaluating pupal exposure to environmental signals under semi-field conditions. To examine if laboratory-reared pupae could undergo emergence in diapause under semi-field conditions, pupae were placed in “mosquito breeder” containers in semi-field conditions during timepoints where larval cohorts were receptive to diapause (**A**). A total of 75 mosquitoes were examined to determine if they emerged in reproductive diapause (**B**). Each dot represents the average follicle size for an individual mosquito, with a 50 µm threshold (dotted line) used to determine individuals in the diapause state. The mean follicle size (+/- SEM) is displayed for each experimental cohort. n, number of individual mosquitoes examined.

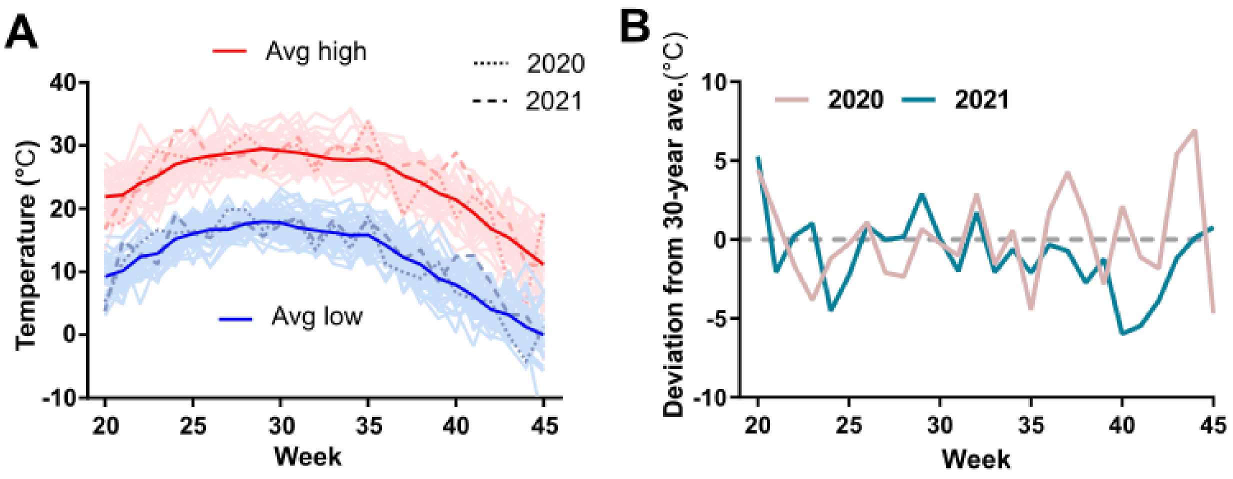

Figure S5. Temperature comparisons of 2020 and 2021 to 30-year averages. To place the temperatures of our two-year (2020 and 2021) semi-field study in context, average high and low temperatures were compared to the historical thirty-year average weekly high and low temperatures in Central Iowa (**A**). The thirty-year average high (red) and low (blue) temperatures are displayed by week, with the average temperatures for individual years displayed by light red (high) or light blue (low) lines. or presented in light red (high) and light blue (low). Average trends for each variable over all years are presented in dark red and dark blue, respectively. The values for 2020 and 2021 are respectfully displayed by dotted or marked lines. (**B**) The deviation in average temperature for 2020 and 2021 is displayed by week from the thirty-year average.

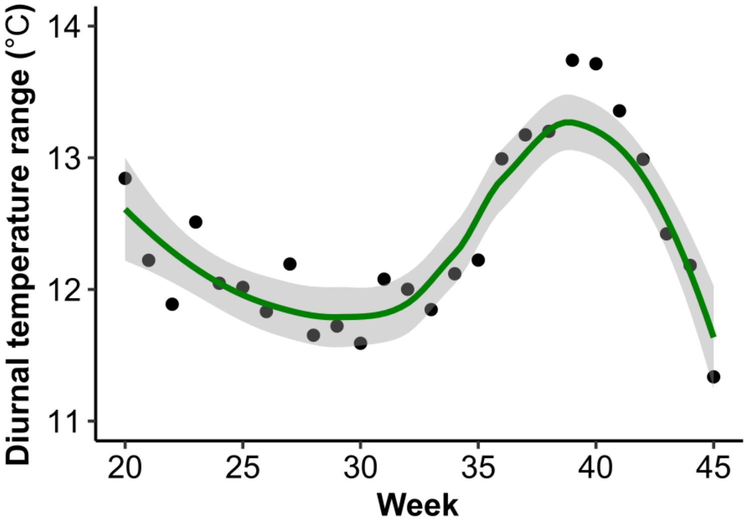

Figure S6. Seasonal changes to diurnal temperature fluctuations. The diurnal temperature range (difference between high and low temperatures) is displayed by week.

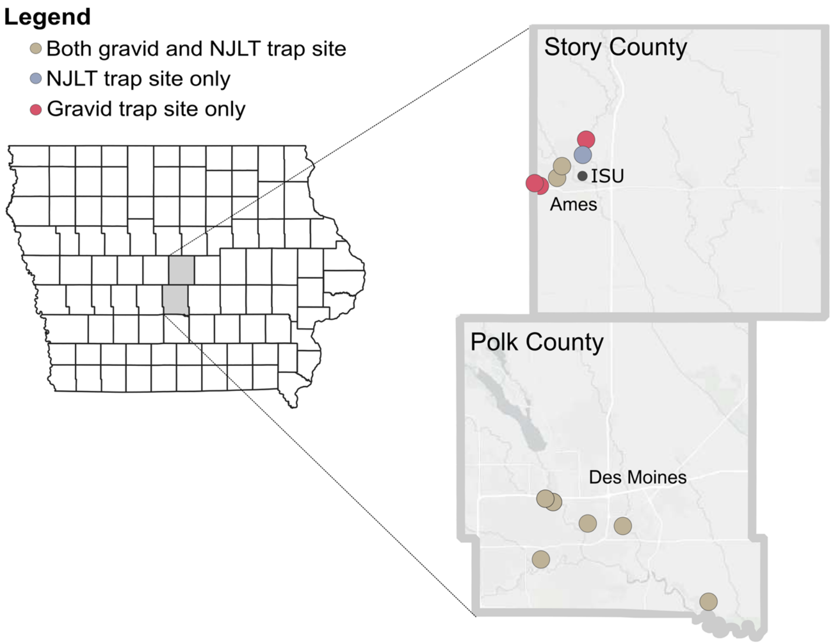

Figure S7. Mosquito surveillance sites in Iowa. Two types of mosquito trapping data were used for this study: Gravid (captures reproductive trends) and New Jersey Light Trap (NJLT; captures general trends). A total of 16 sites were included for their gravid records (Gravid-only sites in red), and 14 sites for their NJLT records (NJLT-only sites in blue) from 2016-2021. The sites denoted in tan provided both gravid and NJLT data.

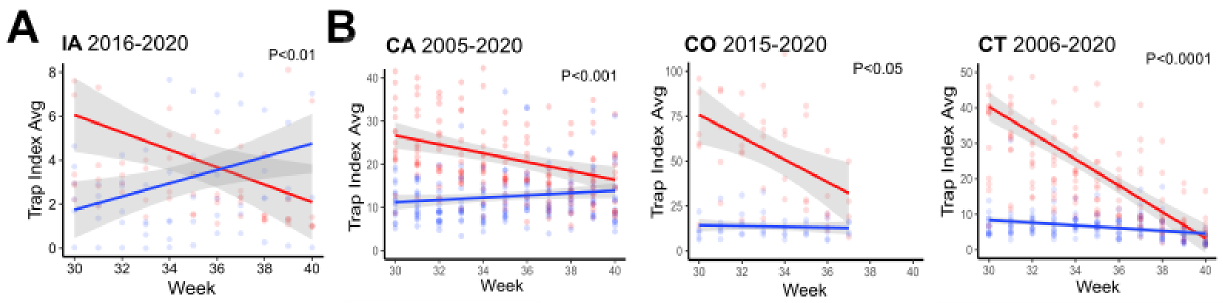

Figure S8. Gravid populations are distinct from general *Cx. pipiens* mosquito abundance. Comparisons of end-of-season gravid trap data (reproductive populations) and New Jersey Light Trap (NJLT; general abundance) for *Cx. pipiens* group mosquitoes in Iowa (**A**) and other locations across the country (**B**). For both **A** and **B**, data display the normalized trap index values from weeks 30 to 40 for the years shown above each graph. Average weekly gravid or NJLT values across all years of trapping are shown by the solid lines (95% confidence intervals in grey), with data from individual years displayed for gravid (light red) or NJLT (light blue) tapping data. The annual slope values for gravid and NJLT trends were compared using a linear regression, with significance indicated for each location.

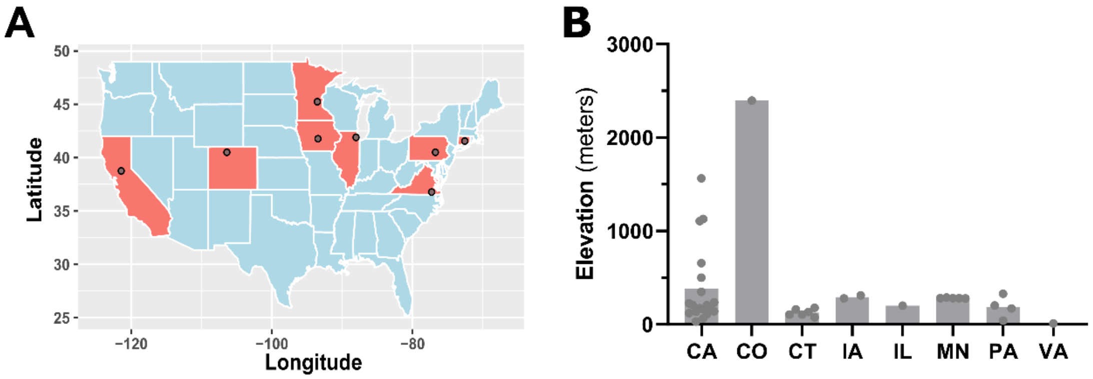

Figure S9. Geographic locations and elevations of trapping site locations across the United States. (**A**) The geographic location (latitude and longitude) of each trapping site location is displayed on the map, with the approximate site location (or centroid of the trapping area) denoted by a circle. (**B**) The average elevation of the participating counties in each state included in our analysis, with individual counties displayed by the grey dots.

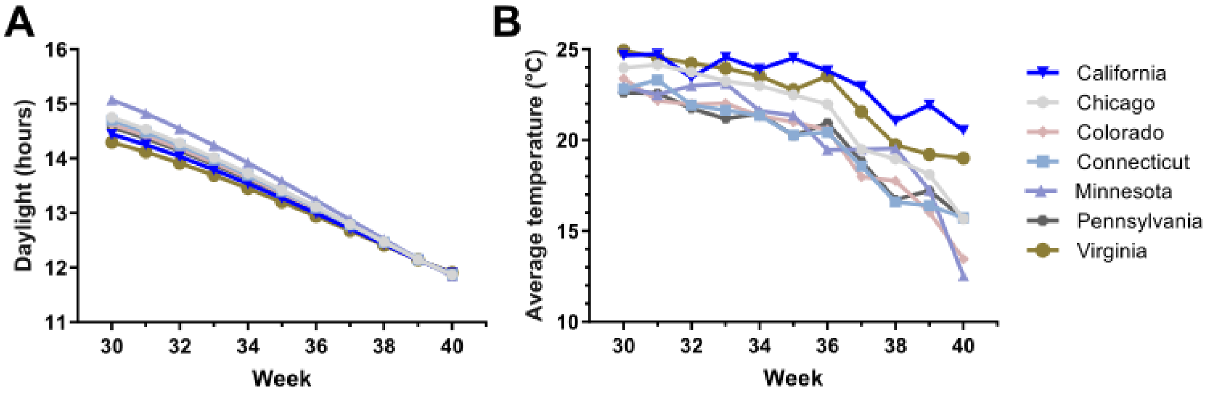

Figure S10. End-of-season temperature and photoperiod conditions at trapping sites across the United States. The average photoperiod (measured as hours of daylight) (**A**) or average temperature (**B**) for each of the locations across the United States.

Table S1*:* Larval *Culex pipiens* semi-field group descriptions, Ames IA.

Year	Start Date	Week Start	Week of first pupae	Week End	Start Temp	Temp at first pupae	End Temp	Start Photo	Photo at first pupae	End Photo	Diapause recorded?
2020	July-19	30	31	33	23.7	19.5	23.5	14.7	14.5	14.0	N
2020	Aug-9	33	34	36	23.5	21.1	18.7	14.0	13.7	13.1	N
2020	Aug-20	36	38	43	18.7	16.2	3.8	13.1	12.4	10.8	Y
2020	Sep-20	39	41	43	18.5	13.8	3.8	12.1	11.4	10.8	Y
2021	Jul-25	30	31	34	23.2	24.9	22.3	14.7	14.5	13.7	N
2021	Aug-15	33	34	35	24.0	22.3	23.9	14.0	13.7	13.4	N
2021	Aug-22	34	35	36	22.3	23.9	20.9	13.7	13.4	12.8	Y
2021	Sep-12	37	39	42	19.4	16.9	14.2	12.8	12.1	11.2	Y
2021	Sep-18	38	39	42	20.3	16.9	14.2	12.5	12.1	11.2	Y
2021	Sep-25	39	41	42	16.9	11.5	14.2	12.1	11.5	11.2	Y
