# Supplementary material for: Semi-field and surveillance data define the natural diapause timeline for Culex pipiens across the United States
Source: Commun Biol. 2022 Nov 27;5:1300. doi: 10.1038/s42003-022-04276-x (PMC9701209; doi:10.1038/s42003-022-04276-x)
Supplement: Supplementary file 3 — Description of Additional Supplementary Files [file 42003_2022_4276_MOESM3_ESM.pdf]

Description of Additional Supplementary Files

File name: Supplementary Data 1

Description: The source data behind the graphs presented in the manuscript.
